# Statistical Analysis of Categorical Time Series of Atmospheric Elementary Circulation Mechanisms - Dzerdzeevski Classification for the Northern Hemisphere

**DOI:** 10.1371/journal.pone.0154368

**Published:** 2016-04-26

**Authors:** Mihael Brenčič

**Affiliations:** 1 Department of Geology, Faculty of Natural Sciences and Engineering, University of Ljubljana, Ljubljana, Slovenia; 2 Geological Survey of Slovenia, Ljubljana, Slovenia; University of Vigo, SPAIN

## Abstract

Northern hemisphere elementary circulation mechanisms, defined with the Dzerdzeevski classification and published on a daily basis from 1899–2012, are analysed with statistical methods as continuous categorical time series. Classification consists of 41 elementary circulation mechanisms (ECM), which are assigned to calendar days. Empirical marginal probabilities of each ECM were determined. Seasonality and the periodicity effect were investigated with moving dispersion filters and randomisation procedure on the ECM categories as well as with the time analyses of the ECM mode. The time series were determined as being non-stationary with strong time-dependent trends. During the investigated period, periodicity interchanges with periods when no seasonality is present. In the time series structure, the strongest division is visible at the milestone of 1986, showing that the atmospheric circulation pattern reflected in the ECM has significantly changed. This change is result of the change in the frequency of ECM categories; before 1986, the appearance of ECM was more diverse, and afterwards fewer ECMs appear. The statistical approach applied to the categorical climatic time series opens up new potential insight into climate variability and change studies that have to be performed in the future.

## Introduction

Weather and climate can be characterised only by a great number of physical parameters (e.g. air temperature, air pressure, humidity etc.) forming a complex picture that is difficult to comprehend. These parameters are characteristic for particular points in atmospheric space, forming extensive scalar and vector fields that consequently result in air mass movements and air distribution patterns. In spite of modern measuring techniques and large data storage capacity, it is not possible to obtain complete information for the characterisation of weather or a climate state at a particular observation time or during a particular time period. Complete characterisation based on the entire set of relevant physical parameters is never possible, and other approaches must be applied to characterise the status of the atmosphere in a particular region.

An alternative approach to this deficiency can be the application of a qualitative or quantitative classification scheme based on predefined classification categories. In such an approach, particular categories represent the state of weather or climate in the whole region, and are defined based on the large set of available quantitative and qualitative data. They transfer multivariate characteristics of weather or climate into one single categorical variable. Each category represents a qualitative summary of all the physical parameters characterising the state of the climate in the given time period.

In the literature, several classifications of climate can be found. They can be divided into three large groups: a) subjective classifications, b) mixed classifications, and c) numerical classifications [1,2]. According to many applications reported in the literature, the most frequently applied approach is subjective classification, which can be established only by a skilful and experienced interpreter who is able to analyse several pieces of available meteorological information defined on the spatial scale (e.g. pressure maps, satellite images, synoptic maps, time series of physical parameters etc.). The weak point of these classifications is the particular level of subjectivity depending on the skills of the interpreter or the group of interpreters; consequently much effort has been expended in establishing efficient numerical classifications. Subjective classifications are interesting because they consist of long data sets, some of which span for more than 100 years; consequently, they represent an important source of data for studying climate variability and change in the 20^th^ and the beginning of the 21^st^ Centuries.

In the literature, various subjective classifications can be found defined at different scales, from local to global hemispherical scale. Only a few classifications exist for the whole hemisphere [1,3]; among them, the best known are the Russian classification approaches of Wangengeim-Girs [4] and Dzerdzeevskii [5,6]. For certain time periods of several decades, catalogues of these classifications on a daily basis have been established and published [7].

Statistically speaking, these classification catalogues represent time series of symbolic values that can be defined as time series of categorical data type or categorical time series. For each time unit–calendar date (e.g. day, week and month) one category is defined and the whole dataset is represented as a sequence of categories given in equal time intervals. Compared to rational data, categorical data only have meaning, but they cannot be ranked, and no algebraic operations (e.g. addition, multiplication etc.) can be performed on them. In the set of categorical data, only counting can be performed and operations related to counts can be executed. In spite of these insufficiencies and lack of information caused by the nature of categorical data, one can ask what the characteristics of the whole categorical time series are. There is also a question of whether on the categorical time series structure analyses, modelling, and forecasting, as in the usual rational climate type time series (e.g. temperature, precipitation time series), can be performed. These questions are especially relevant in studying categorical time series related to climate and weather, where it is well known that rational time series experience various time trends and non-stationarity on shorter and longer time scales. Understanding the relationship between climate variability and climate change is one of the most important present-day scientific challenges; time series of atmospheric circulation pattern categories can represent one possible dataset for studying these relations.

Here, continuous time series of categorical data defining elementary circulation mechanisms (ECM) for the Northern hemisphere, according to Dzerdzeevskii and with the daily interval published by Kononova [7] until 2012 (available at www.atmospheric-circulation.ru) are analysed. Many of the statistical and structural characteristics of the Dzerdzeevskii time series have already been detected in Kononova [7]. Her results are based mainly on counting ECM categories and their frequency determination inside calendar years or months. In these analyses, ECM time series are not treated as continuous but as an assembly of several separate categories. Trends and characteristics detected by Kononova [7] were defined on the annually and monthly counts that were identical to calendar dates.

Here, the ECM classification of Dzerdzeevskii is considered as a continuous categorical time series where, for each day, ECM category is assigned. Our analyses represent an additional and new statistical data treatment to already existing analyses [7]. Contrary to that study, our treatment of data is based on the concept of continuous categorical time series of ECM categories and not on separate categories where their frequencies are observed at sub-periods (e.g. years or decades). Statistical analysis is focused on the relationship between ECM categories in time series where they are understood as a qualitative summary of atmospheric circulation mechanisms. Based on understanding that the ECM classification on a daily basis forms continuous time series of ECM categories, several research questions can be identified and hypotheses defined.

The scope of the paper is twofold. First, we intend to test categorical time series analyses methods on the ECM time series and to detect its structure. In relation to this, the main research questions are: whether a time series is stationary, and whether trends defined as non-periodic and periodic are present. At the same time, new statistical methods for categorical time series characterisation were introduced; randomisation of time series, moving dispersion filters and density diagrams. Secondly, based on the detected structure of ECM time series, changes of atmospheric circulation patterns of the Northern hemisphere during the 20^th^ Century are illustrated. To the best of our knowledge, the methodology presented here has not yet been applied to ECM data or to any other categorical climatic time series. Such an approach has not yet been published.

## Theory and Methods

### Dzerdzeevskii Classification

A short description of the Dzerdzeevskii classification is now presented. For a more elaborate description, see the summary papers in English[8,9], or the original literature in Russian [5–7].

The following description is given after Kononova [7], summarised in Brenčič et al. [10]. The Dzerdzeevski classification characterises the entire Northern hemisphere and trajectories of cyclones and anticyclones over specific regions. From the classification, 41 elementary circulation mechanisms (ECM) are defined. In our analyses, ECM are defined as categories. They differ in direction and quantity of blocking mechanisms and have different numbers of southern cyclone outlets. Each ECM has a unique cyclone and anticyclone trajectory pattern that is described within the classification. ECMs are grouped into 13 types and four groups. The first group is defined as *zoned*; the second defined as *zoned disturbance*; the third defined as *northern meridional*; and the fourth group is defined as *southern meridional*. In the *zoned* group ECM categories *1* and *2* with the anticyclone on the North Pole and with two to four outlets from the south cyclone and the same number of sectors without blocking are included. In the *zoned disturbance* group types *3*–*7* are included. For this group high pressure on the Pole is characteristic, as well as blocking over the entire Northern hemisphere. In the *northern meridional* group, types *8*–*12* are included. For this group, high pressure in the Arctic is characteristic, and two to four blocking mechanisms with the same number of southern cyclone outlets. The *southern meridional* group where only type *13* is present is characterised by cyclone circulation and a front over the Arctic, where at high latitudes southern cyclones protrude into western cyclones. In addition, in the ECM classification, small letters accompany numbers. The small letter *s* stands for summer and *w* stands for winter. The letters *a*, *b*, *c*, and *d* define the geographical location of blocking processes and southern cyclone outlets [8].

In addition to the 41 ECM original categories, two additional blank categories *missing* and *out of type* were added. *Missing* category (HET in the original data series) is defined when the classification for calendar date is missing. Category *out of type* (BT in the original classification) is used when the interpreter was not able to obtain classification in accordance with the Dzerdzeevskii classification. These two blank categories were included in the ECM time series for continuity. It would be better to interpolate these gaps of the ECM time, but no reliable procedure for the replacement exists.

**Fig 1:**
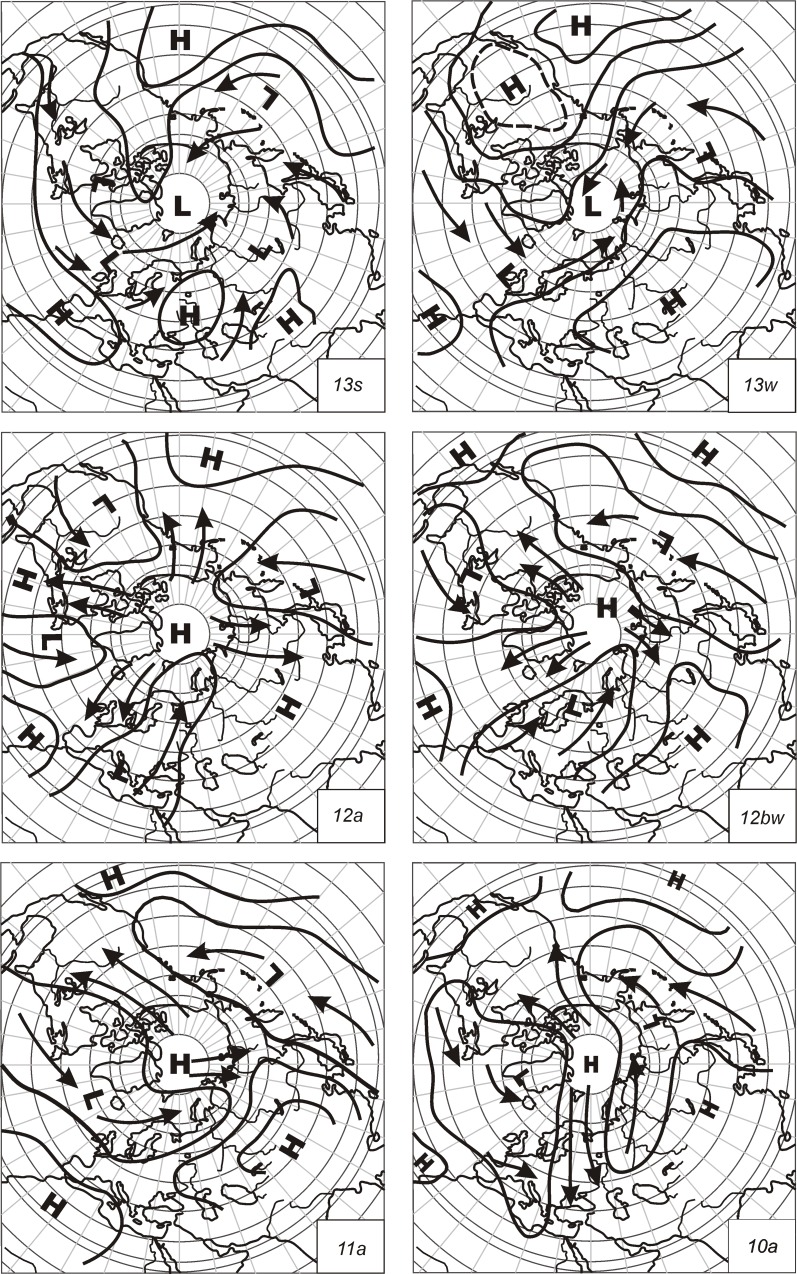
Most frequent ECM maps of the Northern hemisphere (arrows–cyclone trajectory, L–areas of cyclonic activity, H–areas of anticyclone activity) [7].

From the frequency analysis that follows (see Results and Discussion sections), the most frequent ECM categories are: *13s*, *13w*, *12a*, *12bw*, *11a* in *10a*. Short descriptions of their characteristics are given based on Kononova [7] and they are represented graphically in Fig 1.

Processes of ECM type *13* are present during the whole year but with smaller frequency during the winter than the summer. Seasonal changes of this type are the consequence of the changing temperature and pressure field above the continents and oceans. During this ECM, a wide low-pressure depression is formed above the Arctic, which goes into a large part of the extra-tropical latitudes of the Northern Hemisphere. At the same time, two large ocean subtropical anticyclones are developed; in the Western hemisphere, the North Pacific High and over the Atlantic Azores High, which goes towards Western Europe and into the middle of the Mediterranean. ECM *13w* appears mainly from September to May with the highest frequency in December. *13w* is developed as a consequence of a relatively narrow strip of low pressure in the Arctic. Above North America and Asia, extensive areas of high pressure with ridges in the W-E direction are developed. A strong stationary anticyclone is developed over the whole of Eurasia.

All ECMs included in type *12* are characterised by three or four southern cyclone intrusions. In the North Pole, a well developed anticyclone is formed and around the hemisphere several cyclonic series are developed. The movements of these cyclones are parallel to trajectories with a predominant north direction. ECM *12a* is characterised by four Arctic intrusions and four intrusions of cyclones from the south. The positions of these intrusions are not fixed. Among the most frequent Arctic intrusions is the one protruding towards the Atlantic and Western Europe. Between Eastern Europe and Central Asia, a large intrusion of cyclones is present to the north. ECM *12bw* is characterised by three blocking processes and three cyclone intrusions; Arctic intrusions are present in North America, the Atlantic Ocean and Eastern Asia. ECM *12bw* very often follows type *11* when the Arctic cyclone establishes conditions for a third intrusion. It appears mainly from September to May with the highest frequency in February and March.

ECMs of type *11* are characterised by two processes of blocking and three intrusions of southern cyclones. Blocking processes develop above North America and eastern Asia and join above the Arctic; they form the winter basin of anticyclones. For 70% of the time, high air pressure is present above Siberia, and consequently the flow of cold air is developed. With the absence of an extensive stationary anticyclone over North America, an area of high air pressure is developed, enabling the rise of air from the Polar basin towards the south. This is the most representative process of the colder part of the year and includes four variants assigned *a*, *b*, *c* and *d*. Among the most frequent is ECM *11a*. In this case, the Siberian anticyclone covers the entire area of Siberia and the eastern part of European Russia, its core being developed along the river basin of the Lena. At its peripheries, intrusions of cold polar air are present, filling the Siberian anticyclone and influencing its stationarity. On the western part of the globe, the Arctic intrusion influences the central and eastern part of Northern America. Above the oceans, intensive cyclonic activity is present and is related to cyclones on the Arctic front and to the regeneration of polar frontal cyclones that intrude into the systems of the Icelandic, Aleutian and Kamchatka depressions. Intrusions from the south appear in the eastern part of the Mediterranean in Europe and along the eastern shores of North America and Asia. ECM *11a* appears from September to May and is most frequent in January and February.

For ECMs categories included as type *10* two blocking processes and two entrances of southern cyclones are distinctive. Blocking processes are present at the same time above Eastern Europe and Central America, but they differ according to their structure and power in the vertical direction. Intrusions above Europe very often have higher vertical intensity and intrusion above America at the beginning in its low and thin frontal part; in the second half it spreads into higher parts of the atmosphere. Intrusions of southern cyclones flow over America and Far East. In both variants of type *10*, the positions of cyclonic processes are at different places. Processes of this type develop during seasonal changes of air state above the continents, which influences their distribution and persistence. ECM *10a* appears when anticyclones are established above the continents, but not above the oceans. Intensive intrusions above Europe create a stable anticyclone that joins with the Siberian anticyclone. An area of high air pressure is formed above the Arctic and travels towards Eurasia and America. Intrusions above America are represented in the central end eastern part of the continent. In the northwestern part of America, cyclone activity related to the Aleutian depression is developed. The latter fills up with southern cyclones rising along the Far East of Asia. At the same time along the northern part of western Siberia, cyclones related to the narrow depression move and fill up with cyclone intrusions from Kazakhstan and central Asia. Cyclonic activity is also strong in the area of Atlantic Ocean and goes toward the east, covering the western and central part of Europe. At the same time it is connected to the well-developed Icelandic depression that, with polar and frontal cyclones, originates in the temperate zone, developing along the northern coastal area of America. ECM *10a* appears during the whole year with highest frequency in May and April.

### Mathematical Background

#### Preliminaries

Contrary to the classical time series analyses of rational data time series, theory of categorical time series is not fully elaborated. Works on categorical time series are widely scattered among various fields, and practically no systematic monographic descriptions of categorical time series characteristics are known to the author. One exception is the mathematical monograph of Weiß [11]. Newly formulated concepts include moving dispersion filters, density diagrams, and randomisation of time series for seasonality detection. It is quite possible that they are defined under a similar mathematical definition and appear somewhere else in the literature, but at the time of paper preparation this is not known.

#### Categorical time series definition

A categorical variable is defined when *X* takes one of a finite number of unordered categories, say *b*_0_, …, *b*_*m*_. As an example part of our ECM time series can be given: … *13s*, *13s*, *12a*, *13s*, *12a*, *12a*, *11a*, …

The range of *X* is coded as υ = {0, …, *m*}. A categorical random variable with the range υ = {0, …, *m*} has the following probabilistic characteristics

$$P(X=0)=1-\sum_{j=1}^{m}P(x=j)$$ (1)

with marginal probabilities

$$\pi_{i}≔P(X=i)\in (0;1)$$ (2)

and the whole distribution determined by parameter *m* is *π*_1_,…, *π*_*m*_ [12]. A discrete categorical time series is a set of data {*X*_t_: *t* = 1, …, *T*} that is one of possible realisations of the categorical stochastic process (*X*_t_)_ℕ_ The categorical process (*X*_t_)_ℕ_ with the range υ can be represented by binary vectors

$$Y_{t}\in {\left\{0,1\right\}}^{m+1}$$ (3)

with

$$Y_{t,i}=\delta_{b_{i}}x_{t},\;i=0,\ldots ,m$$ (4)

Defining cumulated sums

$$C_{t}≔\sum_{s=1}^{t}Y_{s}$$ (5)

*C*_*t*,*i*_ is number of *X*_s_ where *s* = 1,…, *t*, …, *T* where *T* is total time length of categorical time series *X*_t_ [12].

Empirical marginal probabilities *p*_*i*_ are defined as

$$p_{i}=\frac{n_{\nu }}{T}=\frac{n_{\nu }}{N}$$ (6)

where *n*_*υ*_ is the total number of *b*_*υ*_ observations and *T* is introduced because the total length of the categorical time series is equal to the total number *N* of observations in empirical *X*_*t*_.

#### Stationarity detection

As in rational data time series, stationarity can be the characteristics of a categorical time series. Weiß and Göb [13] define weak and marginal stationarity of categorical time series. Stationarity of categorical time series can be indirectly detected to the rate evolution graph, which is a simple tool for checking marginal stationary. The rate evolution graph of (*X*_t_)_N_ is a multiple line plot of all component series *C*_*t*,*i*_ where all *C*_*t*,*i*_ are plotted simultaneously on one chart. Slopes of *C*_*t*,*i*_ graphs are estimates of corresponding marginal probabilities *π*_*i*_. If (*X*_t_)_N_ is marginally stationary and at most moderately serially dependent, then graphs of *C*_*t*,*i*_ are approximately linear in *t* [11,12]. If at least one *i* of the graphs *C*_*t*,*i*_ is not approximately linear, the whole categorical time series is non-stationary; the greater the number of nonlinear category graphs, the higher the deviation from the stationarity.

#### Location and dispersion measures

For categorical variables, the only possible measure of location is mode *Mo* that in symmetrical distributions of rational data is equal to other measures of location. It is defined such that *p*_*i*_ ≥ *p*_*j*_ for any *i*,*j* in υ and cannot be uniquely defined (e.g. uniform distribution).

For categorical data, various dispersion measures can be used. In social sciences they are known under the common name index of qualitative variation. Among dispersion measures for categorical variables, Weiß and Göb [13] suggest the application of the Gini index, entropy and Chebycheff dispersion. They can be defined as follows:

Gini index

$$\nu_{G}(X)≔\frac{m}{m-1}\left(1-\sum_{i}\pi_{i}^{2}\right)$$ (7)

Entropy

$$\nu_{E}(X)≔-\frac{1}{\text{ln}\;m}\left(\sum_{i}\pi_{i}\;\text{ln}\;\pi_{i}\right)$$ (8)

Chebycheff dispersion

$$\nu_{C}(X)≔-\frac{m}{m-1}\left(1-{\text{max}}_{j}\;\pi_{i}\right)$$ (9)

Empirical dispersion measures are defied alternatively by replacing *π*_*i*_ in the equations above with *p*_*i*_. Intuitively, dispersion measures of categorical variables can be understood as a reflection of the *X* outcome uncertainty. They are defined in the range (0,1). In the case of uniform distribution their value is 1; in this case, intuitively maximal uncertainty about the outcome of *X* is present. In the case of one-point distribution, their value is 0 and intuitively maximal certainty about the outcome of *X* is applicable.

Among dispersion measures, the Gini index is most frequently applied in various fields and has appeared under very different names. Much efforts has been expended in its explanation and understanding. The Gini index is the standardised likelihood of categories in categorical time series falling in the same category. It can be interpreted also as the ratio of variance of expanded binomial distribution to the variance of a binomial distribution [14]. The easiest concept to understand it is as a measure of “unalikeability,” as explained by Kader and Perry [15]. According to this concept, the distribution of categories in the categorical variable is more alike when counts of similar categories are equal or similar compared to the case when counts are very different. From this point of view, uniformly distributed categories have ν_G_ = 1, meaning that counts of all categories are equal. The Gini index can also be understood similarly as the coefficient of variation in rational data [16].

The other two dispersion measures [11, 12] are more difficult to interpret. Studies concerning them are sparse. These two measures have been used as additional indicators that help to discern certain patterns not clearly visible with the Gini index. Entropy ν_E_ is a measure used in information theory and is a function that rises monotonically with *p*_*i*_. As defined in (8), less specific information is available when ν_E_ is close to 1 and more accurate information is available when ν_E_ is close to 0. At the value of ν_E_≈1, information is dispersed among all categories, and at a value of ν_E_≈0 information is concentrated in only one category. The Chebycheff dispersion ν_C_ is based only on the category with maximum frequency and consequently with the highest empirical marginal probability. It does not focus on the probability distribution and its shape, which are somehow included in the other two measures. The inequality ν_G_ ≥ ν_C_ is valid.

#### Moving dispersion filters

With the analogy of moving filters (e.g. moving average, moving median etc.) that are used for noise removal and long-term trend detection in time series, moving Gini index ^M^ν_G_, moving entropy ^M^ν_E_ and moving Chebycheff dispersion ^M^ν_C_ filters were all introduced and are defined as follows.

Moving Gini index

$${}^{M}\nu_{G}(X_{t})=\frac{m}{m-1}\left(1-\sum_{i}^{i+\tau }p_{i}^{2}\right)$$ (10)

Moving entropy

$${}^{M}\nu_{E}(X_{t})=-\frac{1}{\text{ln}\;m}\left(\sum_{i}^{i+\tau }p_{i}\;\text{ln}\;p_{i}\right)$$ (11)

Moving Chebycheff dispersion

$${}^{M}\nu_{C}(X_{t})=-\frac{m}{m-1}\left(1-{\text{max}}_{i}^{i+\tau }\;\pi_{i}\right)$$ (12)

τ is length of the filter window; in our analyses applied as τ = 365 days. *X*_*t*_ is taken at the middle of the filter and half of the categories are before the *X*_*t*_ and half of them after. Moving dispersion filters were used to detect trends and changes in dispersion at the annual level τ.

#### Trend and seasonality detection

In rational data time series analyses, various trend definitions exist. Among the simplest are linear trends and trends defined by nonlinear regression functions. Time series describing natural phenomena are very frequently seasonally dependent. In rational data time series analyses, they are described by periodic functions (e.g. sine and cosine). No such trend detection is possible for categorical time series. However, in the analyses of ECM time series, one can be interested in the appearance of time-dependent change of ECM categories’ frequency, and in the appearance of the seasonality in the long-term pattern of ECM time series. By the definition of Dzerdzeevskii, several ECM categories are seasonally dependent (e.g. summer appearance *7as*, *7bs*, *13s*, *8bs*, *8ds*, *12bs*, *12cs*,; winter appearance *7aw*, *7bw*, *8bw*, *8cw*, *8dw*, *12bw*, *12cw*, *13w*) where the period is annually defined. Therefore, it is expected that annually dependent patterns will appear inside the ECM time series. Consequently, our seasonality analyses were focused on the annual period of 365 days. Other possible cycles remain to be detected with further investigations.

Indirectly trends in the categorical time series can be detected with rate evolution graphs (see the subsection on stationarity). Trends can also be detected with moving dispersion filters (see above). In addition, several other approaches were applied in our analysis. The indicator diagram [17] represents the time-dependent appearance of the particular category indicated as a presence Y_t,i_ = 1 or absence Y_t,i_ = 0. From the density of presences, one can detect time-dependent trends. This can be detected also with the event density *ρ*_e_ defined as

$$\rho_{e}=\frac{n_{i}}{\tau }=\frac{\sum_{i}^{\tau }Y_{t,i}}{\tau }$$ (13)

where τ is observation window, in our case again 365 days. *ρ*_e_ is applied similarly as moving dispersion filters resulting in continuous graph of *ρ*_e_ for a particular ECM category. These graphs are defined as density diagrams.

The appearance of seasonality can be detected on the whole time series or based on the hypothesis of seasonal period with the help of mode. Due to the nature of weather and climate and characteristics of Dzerdzeevskii classification, the expected period of a season is 365 days.

Seasonality based on the whole time series was detected with the empirical autocorrelation function with a similar approach as that applied to rational data time series analyses. For further information on the autocorrelation function, see one of the many textbooks dealing with the topic [18]. The autocorrelation function was calculated on the series where to each of ECM, a non-repeatable random number between 1–43 was assigned. This procedure is called randomisation of categories. Several of such time series were constructed; for each, the empirical autocorrelation function was calculated. They were put on the same diagram and their behaviour was observed to detect seasonality appearance. For detecting the average behaviour of autocorrelation for each time lag, the average was calculated and the average empirical autocorrelation function drawn. At this stage of analyses, our interest is only in the statistical significance of calculated empirical autocorrelations at the annual period of 365 days. Such analysis opens several theoretical questions related to the inversion relations between spectrum, variogram and autocorrelation of time series, but they remain open for further investigations and applications.

Mode analysis was performed based on the annual cycle hypothesis. The ECM time series was divided into periods of 365 days such that {*X*_*t*_: *X*_*y*,1_, …, *X*_*y*,*i*, …,_ *X*_*y*,365_} where *y* denotes calendar years from the start of ECM time series in 1899 to the end in 2012. In our case, mode was calculated for each successive *i* such that *Mo*_*i*_ {*X*_1899,*i*, …,_ *X*_2012,*i*_}. The diagram of successive {*Mo*: *Mo*_*1*_, *…*, *Mo*_*i*_, *…*, *Mo*_*365*_} appearance was drawn to detect seasonal patterns, and the marginal frequency of the ECM categories inside the *Mo* was calculated.

### Calculations

All calculations were performed in a spreadsheet using macro procedures written by the author. The only exception is the calculation of the empirical autocorrelation function performed in the R environment with the function acf in the R package *stats* [19].

### Data Preparation

ECM data are available at www.atmospheric-circulation.ru and for the period between 1899–2008 they are published elsewhere [7]. ECM classes are given for each particular day; all together in the time series starting in January 1899 and ending in December 2012, there are 41,639 data entries, representing 114 years. In the whole dataset, only 31 missing data entries are present. The missing data represent a very small share of the entire dataset but, to maintain continuity of the time series, these data were interpreted as a special category. Following this in the data series there are 43 categories. The original data were checked with various sorting procedures for consistency; typing errors were corrected and data entries were reordered where necessary. Great effort was made to remove inconsistency in data; however original ECM classifications [7] were not questioned. For the scope of this paper, the original ECM classifications given in Cyrillic script were transcribed into Latin script as defined in the classification table of Kononova [7].

## Results

### Descriptive Statistics

**Fig 2:**
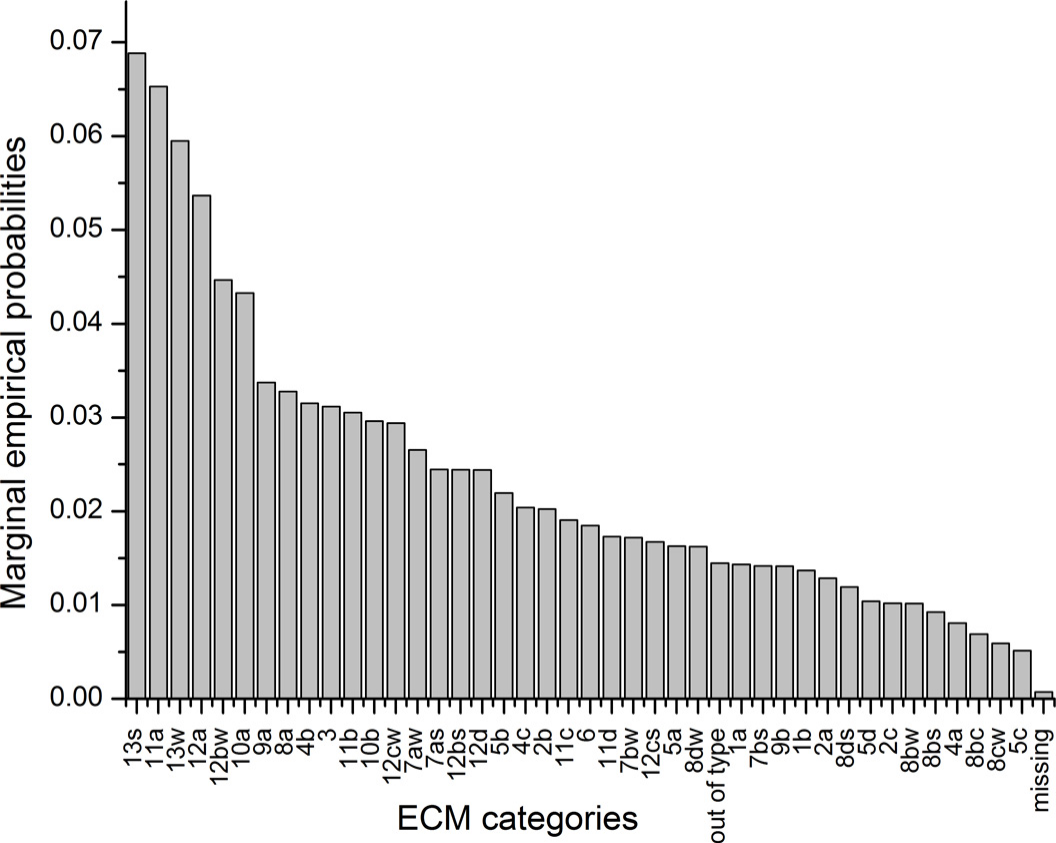
ECM categories sorted by marginal empirical probabilities.

The number of data for each ECM category and their corresponding marginal empirical probabilities are represented in Table 1 and illustrated in Fig 2. In Fig 2, two parts of the diagram are clearly visible. The first is represented by ECM categories *13s*, *11a*, *13w*, *12a*, *12bw* and *10a* and the second is represented by the rest of the ECM categories. Between these two groups, a sharp drop in empirical marginal probabilities is seen. For the other categories, empirical marginal probabilities fall nearly proportionally from category *9a* to category *5c* with the smallest marginal empirical probability. Category *missing* represents a small share at the end of the diagram, showing that it is not part of the patterns and trends in the whole ECM time series. The first larger group of ECM categories is represented by 33.5% of the whole dataset. The most frequent observations are in the category *13s* with a share of 6.9%, followed by the category *11a* with a share of 6.5%.

**Table 1:** Marginal empirical probabilities of ECM categories.

No.	ECM	N	p_i_	No.	ECM	N	p_i_	No.	ECM	N	p_i_
1	*13s*	2866	0.0688	16	*12bs*	1017	0.0244	31	*9b*	589	0.0141
2	*11a*	2719	0.0653	17	*12d*	1016	0.0244	32	*1b*	570	0.0137
3	*13w*	2477	0.0595	18	*5b*	914	0.0220	33	*2а*	535	0.0128
4	*12а*	2235	0.0537	19	*4c*	849	0.0204	34	*8ds*	497	0.0119
5	*12bw*	1859	0.0446	20	*2b*	842	0.0202	35	*5d*	433	0.0104
6	*10а*	1802	0.0433	21	*11c*	794	0.0191	36	*2c*	425	0.0102
7	*9а*	1405	0.0337	22	*6*	770	0.0185	37	*8bw*	423	0.0102
8	*8а*	1365	0.0328	23	*11d*	720	0.0173	38	*8bs*	385	0.0092
9	*4b*	1312	0.0315	24	*7bw*	715	0.0172	39	*4а*	336	0.0081
10	*3*	1298	0.0312	25	*12cs*	696	0.0167	40	*8bc*	287	0.0069
11	*11b*	1271	0.0305	26	*5а*	678	0.0163	41	*8cw*	246	0.0059
12	*10b*	1233	0.0296	27	*8dw*	676	0.0162	42	*5c*	215	0.0052
13	*12cw*	1224	0.0294	28	*out of type*	602	0.0145	43	*missing*	31	0.0007
14	*7аw*	1106	0.0266	29	*1а*	597	0.0143				
15	*7аs*	1019	0.0245	30	*7bs*	590	0.0142				

The overall dispersion measures for ECM time series are: Gini index = 0.99, entropy = 0.94 and Chebysheff dispersion = 0.95.

### Stationary and Trend Detection

**Fig 3:**
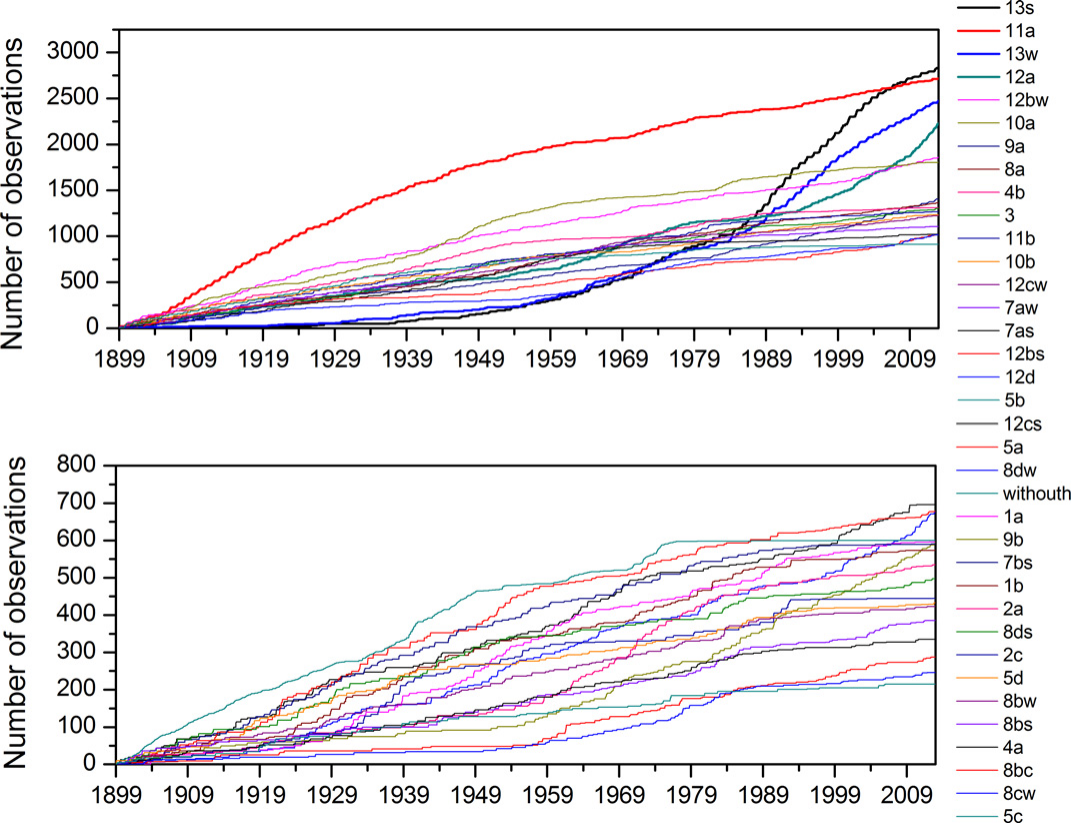
Rate evolution graphs of all ECM categories (for clarity the diagram is divided into two).

In Fig 3, the rate evolution graph of ECM time series is represented by two diagrams; the illustration is divided for greater clarity because a relatively large number of categories is present in the whole time series. The values in the diagram are given as cumulative counts that are proportional to empirical marginal probabilities.

Both diagrams of evolution rate graphs obey the basic supposition of weak stationarity, where it is required that graph with the time evolution should be approximately linear. The shapes of categories with the highest marginal probabilities (Table 1) are convex as well as concave, illustrating that their evolution had changed.

The horizontal curve illustrates no change during the time period (Fig 3); in this period, the category is not present. Such behaviour is typical for categories with small marginal probabilities, showing that they appear only occasionally.

**Fig 4:**
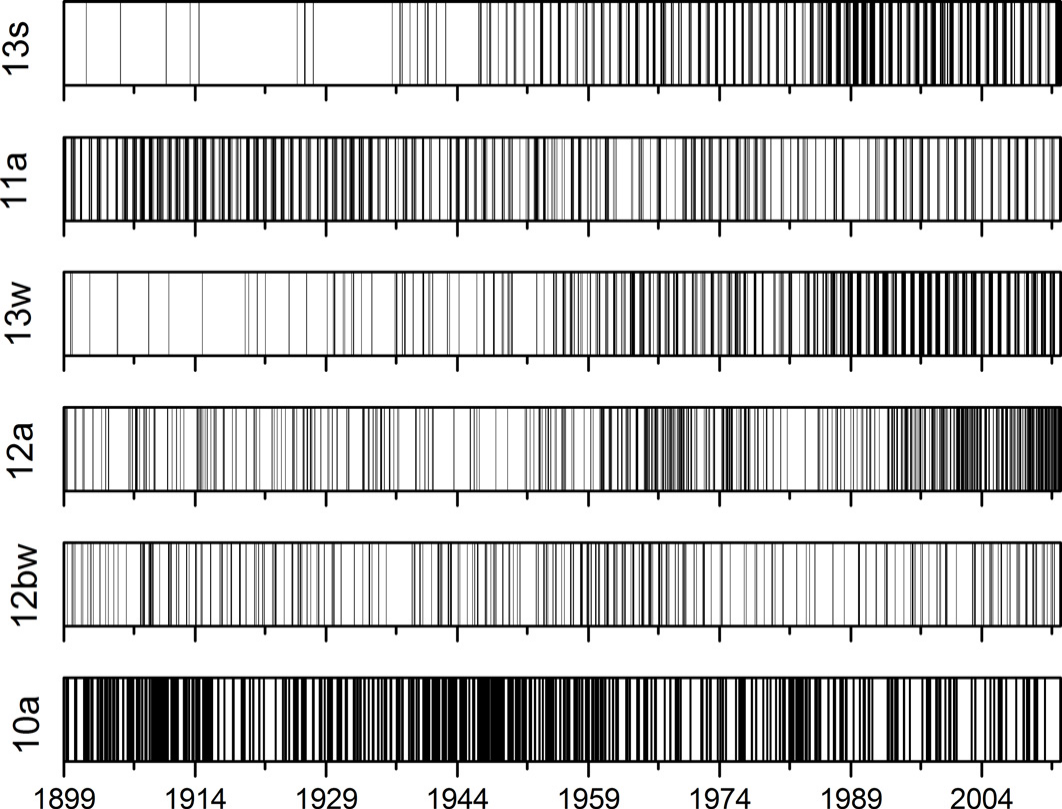
Indicator diagrams for ECM categories with highest empirical marginal probabilities.

**Fig 5:**
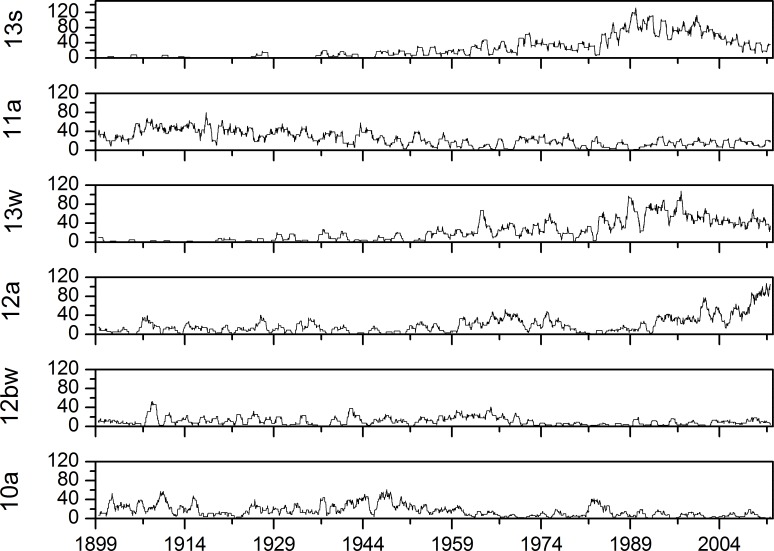
Density diagrams for ECM categories with highest empirical marginal probabilities (window width = 365 days).

Trends in the appearance of ECM categories were observed also on the indicator diagrams represented in Fig 4 and in the density diagram represented in Fig 5, where categories with highest empirical marginal probabilities are illustrated. The window length for density diagrams is 365 days. Diagrams show the non-regular distribution of ECM categories’ time appearance.

### Moving Dispersion Filters

**Fig 6:**
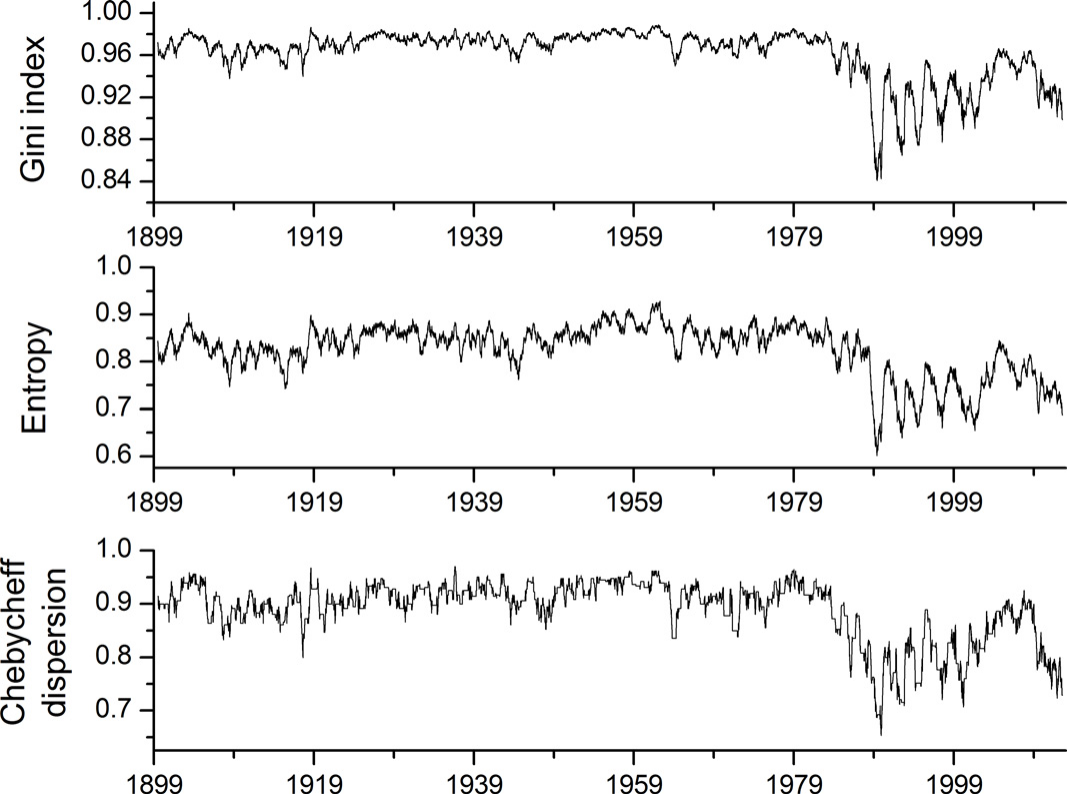
Moving dispersion filters for the window of 365 days (i.e. complete ECM time series).

**Fig 7:**
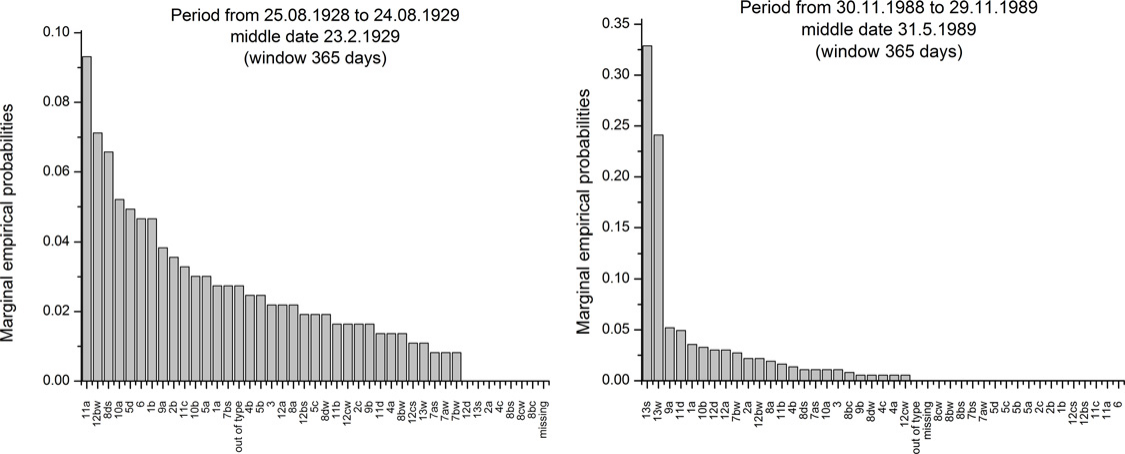
Comparison between ECM categories histograms for 1929 and 1989 (sorted by marginal empirical probabilities).

On Fig 6, moving dispersion filter calculations are represented for the whole ECM time series. From the diagrams, two distinctive periods are apparent. The first goes from the start of the observation period in 1899 until 1986, when the behaviour of the moving dispersion filters changes profoundly. In the first period, fluctuations of the dispersion measures are small; the Gini index is particularly stable. In this period, for the other two measures, fluctuations of values are slightly higher. In the second period from 1986 until the end of the observation period in 2012, the fluctuations of the dispersion measures are much higher. To compare the behaviour of the different periods, two histograms on an annual basis were calculated and are presented in Fig 7. On the left histogram for the period from 25.8.1928 to 24.8.1929 with the median date of 23.2.1929 is represented. This period was chosen as the period when the fluctuation of dispersion measures was small and the Gini measure nearly stable. On the right diagram, the period from 30.11.1988 to 29.11.1989 with the median date of 31.5.1989 is represented where for the median date the lowest dispersion measures were calculated for the whole ECM time series. Differences in the shape of both histograms are apparent. The distribution of sorted counts of categories for the left diagram falls nearly monotonously, and eight ECM categories are missing. The histogram on the right is much more irregular. Categories *13s* and *13w* are much more frequent than the other categories, and 19 ECM categories are missing. The left histogram is close to the triangular distribution where uncertainties of the categories’ appearances are slightly lower than for the uniform distribution. The right diagram approaches more closely with the high frequencies of categories *13s* and *13w* to a point distribution, and has a smaller dispersion as well as a higher certainty for the outcome of particular ECM categories.

**Fig 8:**
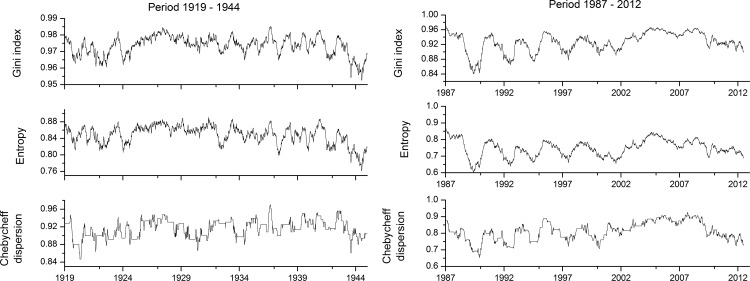
Moving dispersion filters for shorter periods (left period 1919–1944 and right period 1987–2012).

For closer inspection, dispersion measures for distinctive periods were drawn on the diagrams represented on Fig 8. The scales of the left and right diagram are not equal, because the shape of the moving dispersion curve is important. Both curves experience periodicity, however with different amplitudes. They are smaller in the left diagram than in the right diagram. In both cases, the periodicities are on the annual scale and are not persistent across the whole observation scale. On the left diagram, periodicities are observed for the period from 1899–1924 and from 1932–1944. In between, the values of dispersion measures are stable. More distinctive periodicity is observable on the right diagram. However, again, periodicity is not persistent for the whole period and there is a more stable period with slight periodicity starting in 2002 and ending in 2012. There is also a strong negative trend in the moving dispersion curve. The step-like shape of the Chebycheff moving dispersion curve is the consequence of its definition [12].

### Seasonality Detection

Seasonality presence in ECM time series was detected with the empirical autocorrelation function (acf) on randomised ECM categories and with mode analysis. For each observation period, empirical autocorrelation functions were calculated for 20 randomisations and for the maximum time lag of 800 days; slightly longer than two years. In the diagrams, the first lags with much higher autocorrelations are not shown.

**Fig 9:**
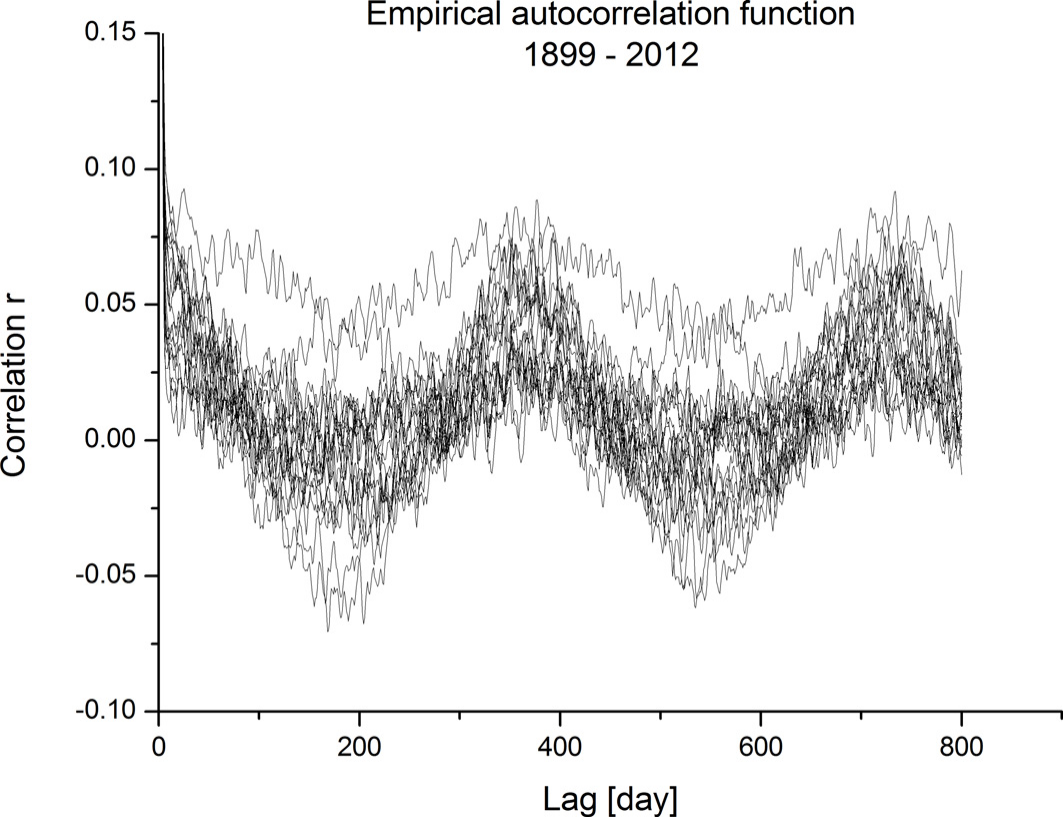
Empirical autocorrelation function for the whole dataset: 1899–2012.

On Fig 9, acf realisations are presented for all randomisations and for the whole observation period from 1899 until the end of 2012. The seasonality pattern is clearly visible with higher correlations at lags of one and two years. Correlations for these lags are significantly greater than zero. In the diagram, some acf are completely out of seasonality pattern and are shifted to slightly higher correlations. It is interesting that all acf positive correlations exist at lags of one and two years, but at lags of 0.5 and 1.5 where negative correlations are expected, the dispersion of correlation values is higher than at the annual lags. At these lags, some correlations are even positive and significantly different to zero.

**Fig 10:**
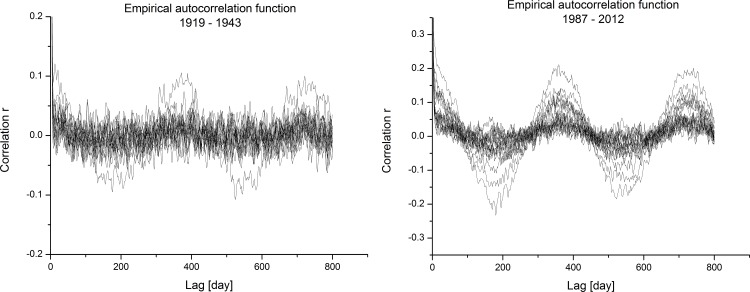
Comparison between empirical autocorrelation functions (left period: 1919; right period: 1987–2012).

Realisations of acf were calculated also for sub-periods detected with moving dispersion filters (Fig 8) and illustrated in Fig 10. The left diagram is for the period of 1919–1943 and the right diagram is for the period of 1987–2012. The differences are clearly visible between both diagrams. For the left diagram, the seasonality pattern and its periodicity are hardly observed; only some discrepancies as expected with randomisation procedures are observed at lags of one and two years. A much more clear seasonality pattern is observed for the latter period on the right diagram. Higher correlations at lags for one and two years are clearly visible; some of them are relatively high. In this case, also negative correlations at lags of 0.5 and 1.5 years are detectable, which is the seasonal pattern usually expected in climatic variables defined on the rational scale (e.g. time series of daily temperatures). Comparison between these two diagrams confirms observations from the moving dispersion filters, where the amplitudes of periodicity observed were smaller in the earlier period than in the later period.

**Fig 11:**
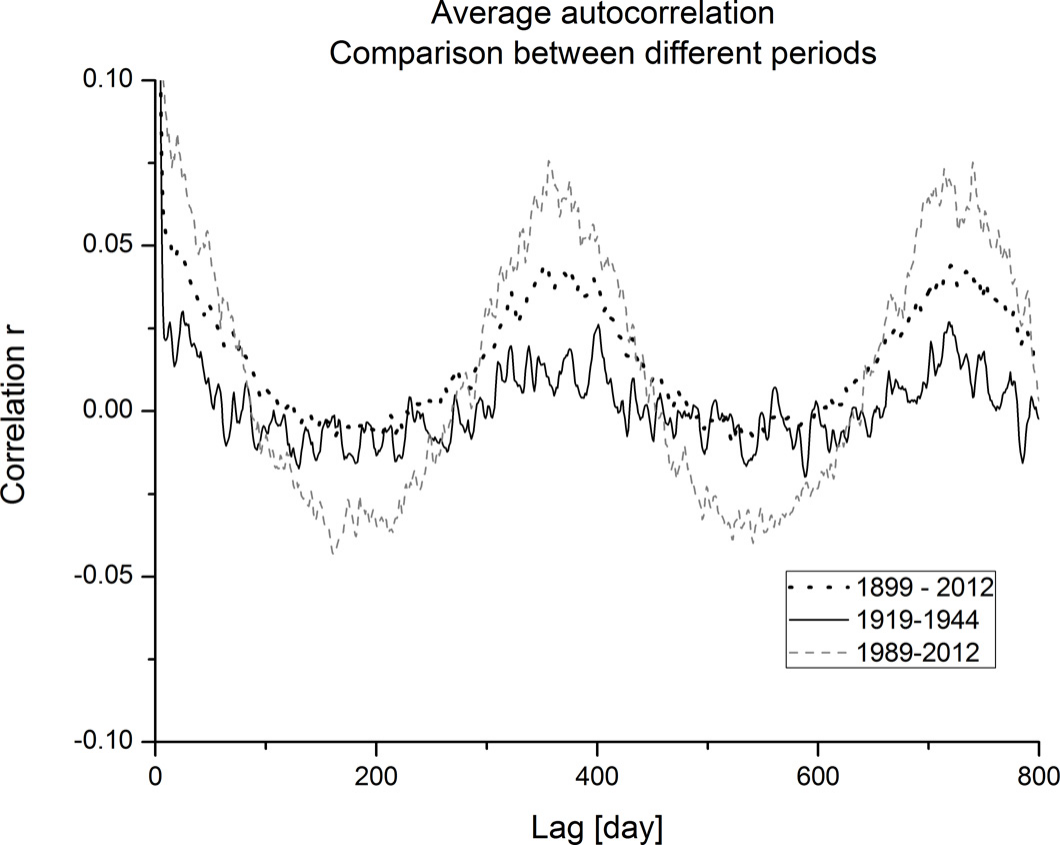
Average autocorrelation–comparison between different observation periods.

With the attempt to clear out the average seasonal acf behaviour, average acf are shown in Fig 11. Average acf curves are different as expected from the diagrams before (Figs 9 and 10). The smallest correlations are detected for the data period from 1919–1944. In this case, the acf curve highly fluctuates around zero; only slight changes are present at lags of one and two years, showing weak periodicity. The curve for the whole data period from 1899–2012 has acf with more clear seasonality pattern at lags of one and two years but at lags 0.5 and 1.5 years, correlations are zero. The most profound seasonality pattern is visible for the acf of the period of 1987–2012. High correlations are visible at lags of one and two years; at lags of 0.5 and 1.5 years, correlations are negative. Comparison between all three acf curves confirms observations from moving dispersion filters, where a different level of periodicity was observed along the whole ECM series and where periodicity in certain time periods appears and disappears, related to different time-dependent dispersion of ECM categories.

**Fig 12:**
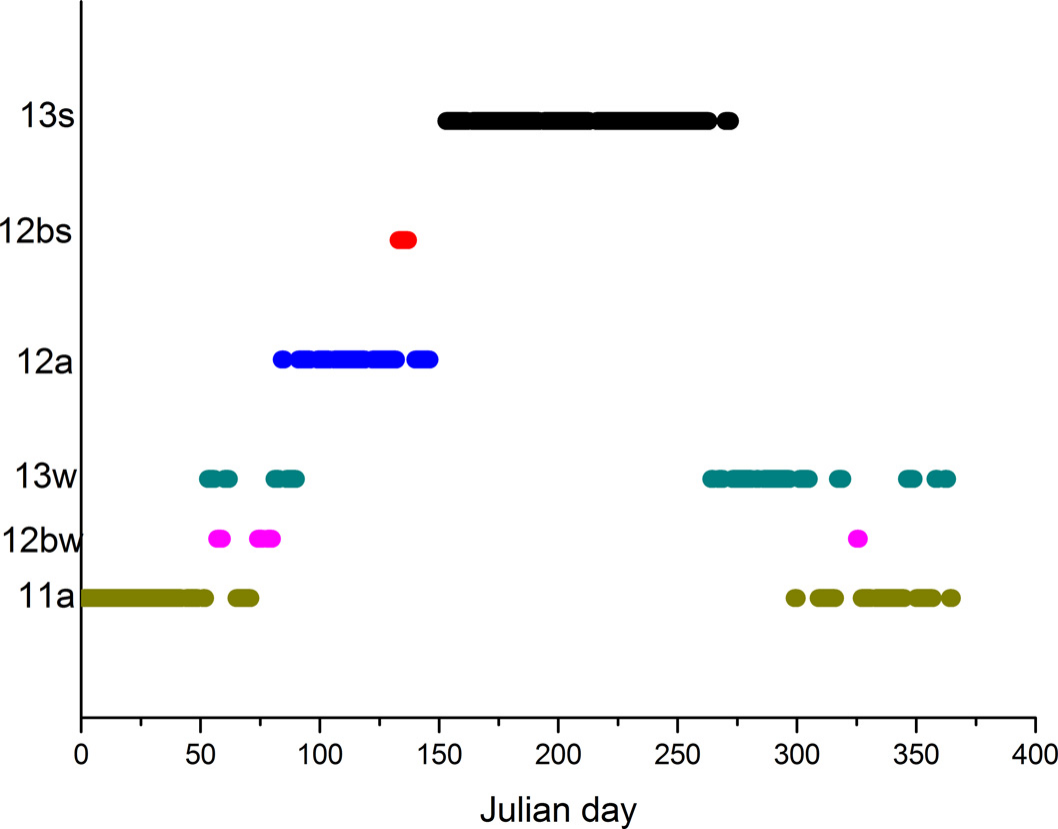
Modus of ECM categories for Julian day (only shown: categories with p_i_ >0.015).

By mode analyses for each of the Julian days, modus were detected. They are represented on Fig 12 where only those modus categories with higher than 0.015 marginal probabilities are illustrated. Marginal empirical probabilities on an annual cycle are also illustrated in Table 2. The most frequent modes are the same ECM categories as those in the whole time series. They are clearly distributed by the seasonal pattern. Summer ECM category *13s* appears as a modus from the beginning of June until the end of September. ECM category *11a* appears in winter from the end of October and until the end of February. In this period, category *11a* is replaced sometimes by *13s* and rarely by *12bw*. For spring months, modus is in *12a* category. Irrespective of how many categories in modus diagram are vertically distributed, the seasonality pattern is clearly visible.

**Table 2:** Marginal empirical probabilities on an annual cycle.

ECM	N	p_i_
13s	112	0.3068
11a	102	0.2795
13w	62	0.1699
12a	48	0.1315
12bw	15	0.0411
12bs	6	0.0164
3	5	0.0137
4b	5	0.0137
11b	2	0.0055
10a	2	0.0055
1a	2	0.0055
12cs	1	0.0027
1b	1	0.0027
5b	1	0.0027
9a	1	0.0027

## Discussion

### ECM as Categorical Time Series

Dzerdzeevskii and co-workers [5,6] defined 41 ECMs with which they classified elementary circulation mechanisms of the atmosphere in the Northern hemisphere. In addition, the category *out of type* when it is not possible to apply one of established ECMs is defined. Frequency analysis has shown that *out of type* represents only a minor part of the whole dataset (Table 1) and, consequently, information about atmospheric circulation in the period between 1899–2012 can be understood as continuous. At the same time from the *out of type* frequency, it follows that within the ECM classification approach nearly all possible patterns of atmospheric circulation for the Northern hemisphere are included.

If classification is given for each particular calendar day and time series of these classifications is formed, then it is possible to observe how different ECMs are distributed in time, how they are related to each other, and how the time series behaves as a whole. One may ask whether relations between different ECMs are time-persistent or whether they change during the course of time such that the time-dependent behaviour of the whole ECM time series changes.

In spite of the fact that ECM time series is categorical, several characteristics of time series as in the case of rational time series can be detected. As is the case in rational time series where a particular time series is one of the realisations of a stochastic process, the same can be understood in the case of categorical time series; first is the realisation of rational stochastic process, and the second is the realisation of the categorical stochastic process. This process is usually understood as a model of physical processes or phenomena. When this process or phenomena are reflected and realised in a particular rational time series (e.g. average daily air temperature, daily precipitation, average daily wind speed etc.) with statistical analyses, their various characteristics can be observed and detected. Such characteristics of the time series are descriptive statistics, frequency analyses, presence of stationarity and non-stationarity, ergodicity, and trends; whether they are periodic or non-periodic. With a similar approach, categorical time series of ECM can be observed. However, in this case, conceptually different processes are observed, as is the case with rational time series where physical parameters are observed in one measuring point or they are assembled over certain areas. The ECM time series represents the state of atmospheric circulation patterns across the entire Northern hemisphere at once on a particular day. Furthermore, the particular ECM reflects and summarises the whole set of all possible physical parameters that have consequence in the certain atmospheric circulation pattern. Certain ECMs summarise all information about atmosphere circulation characteristics on the particular day for the entire Northern hemisphere. With the observation of ECM time series, we indirectly observe how the entire assembly of parameters influencing atmospheric circulation patterns changes during the course of time.

The atmosphere is dynamic system and therefore it is expected that ECMs will alternate in time. If this alternation and time pattern of consecutive ECMs is stable, this reflects stable hemispherical atmosphere circulation; if the patterns of ECM sequences change with time, hemispherical atmospheric circulation is not persistent.

### Distribution Analysis of ECM Categories

The categorical histogram (Fig 2) and frequency counts (Table 1) of the entire ECM time series illustrate unevenly distributed ECM categories; some are more frequent than others. This results in the histogram having a triangular shape and is also reflected in the high dispersion measures. High dispersion measures are usually the result of the uniform distribution, but cursory experimental simulation shows that for the triangular distributions, dispersion measures are also high and positioned in the interval between 0.9–1.0. When the differences among marginal probabilities are small, as in our case, the values of dispersion measures are closer to the value of 1.0 than 0.9. From the theory of dispersion measures, it follows that for ECM time series, the uncertainty expected for each of the categories is high and that ECM categories are [15] very much alike.

From the descriptive statistics, it follows that 33.5% of the entire ECM time series is represented by only six ECMs and that the marginal empirical probabilities illustrated in Fig 2 form two groups. This group profoundly influences stationarity and trends of the entire ECM time series. The non-stationary behaviour is reflected in the rate lines of ECM categories in the rate evolution diagram (Fig 3). Lines of ECMs with highest marginal probabilities are predominantly convex as well as concave. Such lines illustrate that the time evolution of these ECMs has different rates. Convex shapes show that at the beginning of observation period they were relatively frequent, but at later periods their number started to decline. A typical representative of such behaviour is the rate curve of category *11a* which was until the middle of 1980s the predominant category in ECM time series. On the contrary, the concave curves illustrate that at the beginning their frequency was relatively small, but improved during the course of time. Typical representatives of such behaviour are the curves of ECM categories *13s*, *13w* and *12a*, which start to predominate in the second half of the 1980s.

In same cases rate evolution graphs are nearly linear; however this does not mean that they reflect some kind of the stationarity. If one of the categories in the whole categorical time series experiences a convex or concave shape of the rate evolution graph, the series is non-stationary. Due to the continuity of time series change in the rate of one category, it also causes the change of the rate in other categories. From the rate evolution graphs, it can be concluded that time series of ECM categories are non-stationary with strong time-dependent trends.

Relations between most frequent ECMs are also illustrated in Fig 4 with the indicator diagram and in Fig 5 with the density diagram. At the start of the observation period, the ECM categories of *13s* and *13w* appeared only occasionally and their density was nearly negligible. The appearance of category *13s* is less frequent than category *13w*. Their densities start to rise in the middle of 1940s and then their density appearance is similar until the period 1980–2000, after which it slightly decreases. Category *11a* shows a higher appearance at the beginning of the observation period and its density decreases slowly through the whole observation period. Similar time-dependent behaviour is observed with the category *12bw;* however it is slightly more regular than category *11a* time dependence. Grouping in short time periods is observed for category *12a*, which shows higher densities in 1960s, then decreases and abruptly starts to rise from on the 1990s onwards and is very high–the highest among all categories at the end of the observation period. Again, different behaviour is observed for category *10a*. Grouping of events is again observed at this time from the start of the observation period until 1920 and again in the period between 1930–1960. For category *10a* again short grouping is observed around 1980. For this category, short time grouping of the events is distinctive.

From the observations with the window length of 365 days, it follows that two types of trend behaviour are present. The first is a monotonic time-dependent trend, which increases as in the case of categories *13s*, and *13w* or decreases as with categories *11a* and *10bw*. The second is density growing on the short timescale, reflecting grouping of the category in the certain time period; this is characteristic for categories *12a* and *10a*. Similar categorisation of time-dependent trends detected with observations of indicator diagrams and density diagrams can be detected also for other categories with smaller empirical marginal probabilities that are not shown in Figs 4 and 5, but are not as important as the first six ECMs and are therefore not listed.

### Time-Dependent Changes

The time-dependent pattern of ECM time series depends not only on the most frequent ECM, but also on the whole set of categories in the given time period. This is illustrated with moving dispersion filters (Fig 6) and with closer investigation of distribution diagrams for certain periods (Fig 7). Changes in ECM time series structure are the result of time-dependent processes of atmospheric circulation and are reflected in different time-related appearances of certain ECM categories.

The behaviour of moving dispersion curves shows the appearance and disappearance of the periodicity in the air circulation pattern of the Northern hemisphere. At some periods the behaviour of the air circulation pattern is periodic. In winter months, dispersion measures are higher, showing similarity with the triangular empirical distribution of ECM categories; in summer, dispersion measures are smaller, showing empirical distributions more close to point distribution. For other periods when the dispersion measures are higher, no periodic behaviour is present in the time series. This means that during these periods, the empirical distribution of ECM series is close to the triangular distribution model. The appearance and disappearance of periodicity can be explained by the relation between ECMs, which is according to their characteristics seasonally dependent (e.g, those with marks *w*–winter and *s*–summer) and ECMs that appear during the whole year. When periodicity is clearly expressed, seasonally dependent ECMs prevail, and where there is weak seasonality present or where ECMs do not appear for the whole year, they are dominate over seasonally dependent ECMs.

Based on the ECM classification, Kononova [7,9] separates the total available record between 1899–2012 into three distinctive epochs. Her classification is based on counting procedures and on expert interpretation of the available record. In the first epoch between 1899–1916, northern meridional processes are predominant. In the second epoch between 1916–1956, zoned processes are predominant; and in the last epoch from 1957 until the present, the predominant processes are southern meridional. This final epoch is further divided into four sub-epochs. The first is defined between 1957–1969 and is characterised by a simultaneous increase in duration of northern and southern meridional processes. The second sub-epoch between 1970–1980 is characterised by an increase in the duration of zoned processes. In the third sub-period between 1981–1997, a prolonged duration of southern meridional processes is present. In the final sub-epoch between 1998–2012, the duration of meridional southern processes is shortened and that of meridional northern processes is prolonged.

In comparison to conclusions from Kononova [7,9], from our statistical analysis similar conclusions cannot be reached. The milestones defined by her cannot be identified in our analysis. From our calculations, it follows that those most frequent ECMs that have the highest weight on the dispersion measures calculations are related only to meridional circulation. ECM *11a*, characterised by the northern meridional circulation, was more frequent at the beginning of 20^th^ Century. At the end of 20^th^ Century ECMs *13s* and *13w* that have southern meridional circulation are predominant. In the recent years, the frequency of *12a*, again northern meridional, is increasing. ECMs characterised as zoned are less frequent and subordinate and therefore do not influence the dispersion measures. In the entire time series, the most frequent zoned ECM is *4b*, counting only the ninth most frequent among all categories (Table 1).

The most important milestone that can be discerned from our calculation of dispersion measures (Fig 6) is the year 1986, which is positioned between Kononova’s sub-epoch of 1981–1997 when (according to her) southern meridional processes are predominant. Until 1986, dispersion diagrams (Fig 6) do not show significant differences. A significant change in dispersion measures appears after 1986. Between 1986–2012, the ECM time-dependent pattern profoundly changes. On a short time scale, fluctuations of dispersion measures become profound, and change from low to high values, indicating that the distribution of ECM is no longer stable and changes on relatively short time periods. In the period after 1986, ECM time series become less diverse with the appearance of a smaller number of ECM categories. Before 1986, relations between different ECMs are persistent; after this year, they changed and were no longer stable. In this latter period predominant ECMs have much higher frequencies than other ECMs with lower frequencies, and the distribution of ECMs became more of a point shape distribution than before 1986, when its shape was more triangular. In the period after 1986, the behaviour of atmospheric circulation abruptly changed, and the diversity of different circulation patterns diminished when some kind of grouping of ECMs appeared.

If ECM categories are a reliable description of elementary circulation patterns, such a sharp change in dispersion measures behaviour illustrates a profound change in atmospheric circulation. Before 1986, the atmospheric circulation was more diverse with many more elementary circulation patterns present than after that year, when the frequencies of certain ECMs diminished significantly or even completely disappeared. In spite of the fact that moving dispersion filters and periodicity diagrams are not measures of chaotic behaviour, it seems form moving dispersion filters that the ECM time series after 1986 becomes more chaotic, and that the number and frequency of ECMs were no longer stable but changed with time. This is an important conclusion pointing to changes in global climatic regime.

## Conclusions

The concept of categorical time series analyses seems promising in studying time series of climatic categories, as illustrated by the Dzerdzeevskii classification of Northern hemisphere elementary circulation mechanisms. However, more detailed and thorough study is needed in the future. The research should be geared towards two directions. The first should be directed towards establishing a mathematical background of categorical time series analyses for application in practical studies. In the second direction, structural characteristics of ECM time series must be related to physical processes. At first sight, categorical time series analysis is perhaps not as important as time series analysis of other climatic time series (e.g. air temperature data). However, for rational climatic parameters time-dependent trends, periodicity and non-stationarity are their important characteristics helping to understand climatic behaviour. The same can be true also for categorical ECM time series; this study represents the first step in this direction. In our research we have tackled some general explanations of the underlying processes, but more work is needed. A wide field of investigation opens also on other classifications similar to Dzerdzeevskii and their time series. The approach presented here offers quantification of the whole ECM time series regardless of categories, and therefore represents a possible way to study relations with other climatic parameters of rational data type, as well as a comparison to other climatic-dependent phenomena.
